# Research on Substation Electrical Proximity Early-Warning Technology Based on the “Electric Field + Distance” Double Criterion

**DOI:** 10.3390/s25123761

**Published:** 2025-06-16

**Authors:** Jing Zhao, Shengfang Li, Qianhao She, Wenyan Gan, Xian Meng, Qian Wang, Yingkai Long, Qing Yang, Jianglin Zhou

**Affiliations:** 1 State Grid Chongqing Electric Power Research Institute, State Grid Chongqing Electric Power Company Ltd., Chongqing 401123, China; 2 National Key Laboratory of Power Transmission and Transformation Equipment Technology, School of Electrical Engineering, Chongqing University, Chongqing 400044, China

**Keywords:** MEMS sensor, ultrasonic sensor, double criterion, electrical proximity early warning

## Abstract

With the continuous improvement of China’s power grid, safety issues in substation operation and maintenance have become increasingly prominent. However, the existing electrical proximity early-warning devices are inadequate for the complex environments of substations, highlighting the urgent need to develop new electrical proximity early-warning technologies. Based on the safety needs of substation operators, this paper proposes an electrical proximity early-warning method that integrates ‘electric field + distance’. It combines MEMS electric field test technology with ultrasonic ranging technology and designs a double-criterion electrical proximity early-warning device. Based on the COMSOL 6.0 finite-element electric field simulation and the construction safety specification for substation equipment, a multistage electric-field early-warning threshold has been reasonably formulated. A field test conducted at a 220 kV substation demonstrates that this device can issue alerts for various electrical proximity threat levels of the circuit breaker within 0.1 s, which is faster and more accurate than existing commercial electrical proximity early-warning devices. The double-criterion early-warning system minimizes the risk of missed alarms during multi-distance measurements. Additionally, its flexible warning threshold accommodates the increasingly complex operational requirements of substations.

## 1. Introduction

With the continuous growth of national industrial and economic strength along with the continuous expansion of power systems, the safety of power production operations, such as construction, operation, and maintenance, has become increasingly important, given the central role of power systems as a hub [1,2].

At present, substation operators usually install temporary fences or protective nets [3,4] before these operations. However, these basic protective measures do not effectively isolate the area, and operators can easily enter hazardous electrical proximity areas [5] without further warning. In addition, when lifting machinery is operating beside a high-voltage line, there is also a risk of improper operation and inadvertent contact with the high-voltage transmission line [6,7]. Such accidents can result in severe casualties and lead to the tripping of transmission equipment, ultimately having a profound impact on the daily lives of residents [8]. Therefore, the development of electrical proximity warning technology tailored for substation operators and construction machinery is of paramount importance [9,10].

In recent years, electrical proximity warning devices have been developed both domestically and internationally to a significant extent. However, most of these devices rely solely on a single feature quantity as the electrical proximity criterion.

A common electrical proximity early-warning scheme utilizes the distance feature quantity as the safety criterion for electrical proximity. For instance, the HDGJ-I high-power alarm device produced by Baoding Huadian Electric Co., Ltd., Baoding, China, [11] triggers an alarm signal when the distance between any part of the working vehicle and the high-voltage line falls below the safe threshold. LEICA, Wetzlar, Germany, has developed a touch-resistant system [12] that restricts the maximum height of the excavator to prevent electric shocks from construction machinery. Kimio Yamada from Japan proposed a method for identifying high-voltage transmission lines using infrared detection and image processing technology, which involves measuring the distance from the high-voltage charged body [13]. However, this type of electrical proximity sensor, which relies solely on distance as a safety criterion, lacks robustness in complex environments. Early-warning methods based on the principle of laser ranging are susceptible to climatic conditions, and their reliability diminishes in environments characterized by rain and fog [12]. And early-warning technologies that utilize positioning and trajectory tracking necessitate complex base station construction, which complicates the accurate definition of safety boundaries under varying operational states of substation equipment [14].

In addition, some companies have also tried to use electric field strength as the electrical proximity criterion. The Institute of Electronics at the Chinese Academy of Sciences has developed an electric field measurement and early-warning system based on MEMS technology. This system issues continuous alarm signals when the safety threshold of the electric field is exceeded, alerting the operator to potential dangers [15]. The Kanda Communication Company of Japan, Tokyo, Japan, has developed a watch-type electrical proximity alarm [16]. When the operator wears this alarm and inadvertently enters a live area, it emits a continuous audible alarm signal promptly, reminding the operator to be aware of the potential danger. This type of electrical proximity early-warning device establishes the electric field alarm threshold in advance and subsequently achieves near-field early-warning functionality through real-time detection of electric fields. However, relying solely on electric field strength as a criterion is inadequate for evaluating multi-conductor coupled electric fields. The intensity of the electric field is prone to interference from the electromagnetic fields produced by substation equipment, leading to substantial errors in alarm distance that can reach up to the meter level [17]. Furthermore, the absence of miniaturized electric-field sensors that offer high measurement accuracy and stable performance in wearable early-warning devices restricts the research and development of this technology [18]. Traditional parallel pole plate sensors have measurement distortion problems affected by edge electric field distortion [19], while new types of sensors such as MEMS are primarily in the experimental stage and lack insufficient on-site testing.

To sum up, most current electrical proximity warning devices adopt a single-criterion electrical proximity warning technology; such devices often cause inaccurate electric-field or distance measurements due to the complex field working environment, making them prone to false alarms and leakage alarms.

In summary, most current electrical proximity warning devices utilize a single-criterion electrical proximity warning technology. However, such devices often yield inaccurate electric field or distance measurements due to the complexities of the working environment, which makes them susceptible to false alarms and leakage alarms.

This paper proposes a method for substation warning based on a double criterion of “electric field + distance”, derived from the measurement principles of a MEMS electric-field sensor and a piezoelectric-type ultrasonic distance sensor. It designs a du-al-criterion overrun early-warning framework that integrates both electric field and distance measurements. Through COMSOL simulation and field-measured data, the study establishes the warning threshold for substation equipment, culminating in the development of a prototype electrical proximity warning device and an evaluation of its warning effectiveness.

## 2. Double Criterion of Electric Field-Distance Sensor Measurement Principle

### 2.1. MEMS Electric Field Measurement Principle

The MEMS sensor achieves an indirect measurement of the voltage field by measuring the contactless rate of change of the electric displacement vector D’ [20]. Regarding the equivalent charge method, it can be demonstrated that the electric field around the charged conductor has a linear relationship with its potential. Taking the bus in the substation as an example, the relationship between the surrounding electric field and the conductor under the boundary condition of Formula (1) is illustrated in Formula (2):

(1) $$\left\{\begin{array}{l} \varphi_{R\leq r_{0}}=\varphi_{0}(t)\\ \varphi_{R\to \infty }=0 \end{array}\right.$$

(2) $$\vec{E}(t)=\frac{\varphi_{0}(t)}{R\ln(r_{0})}.{\vec{e}}_{R}$$

where $\vec{E}(t)$ is the electric field strength of the measured point; R is the distance between the measured point and the center of the bus; $r_{0}$ is the radius of the bus; and $\varphi_{0}(t)$ is the potential of the measured bus.

When the measuring electrode is placed near the measured conductor, the electrode is coupled by the electric field to produce a changing induced charge, forming a current flowing through the load resistance connected to the electrode, the induced charge *Q* can be obtained by Gauss’s theorem, and the external electric field strength at the electrode is proportional:

(3) $$Q=\varepsilon_{0}A_{eq}E(t)=\frac{\varepsilon_{0}A_{eq}\varphi_{0}(t)}{R\ln(r_{0})}$$

When the changing charge forms a current flowing through the load resistance connected to the electrode, the relationship between the sensor output voltage *U* and the rate of change of the measured potential *φ* is as follows:

(4) $$U_{0}=R_{m}\frac{dQ}{dt}=\frac{\varepsilon_{0}A_{eq}R_{m}}{R\ln(r_{0})}\frac{d\varphi_{0}(t)}{dt}$$

**Figure 1:**
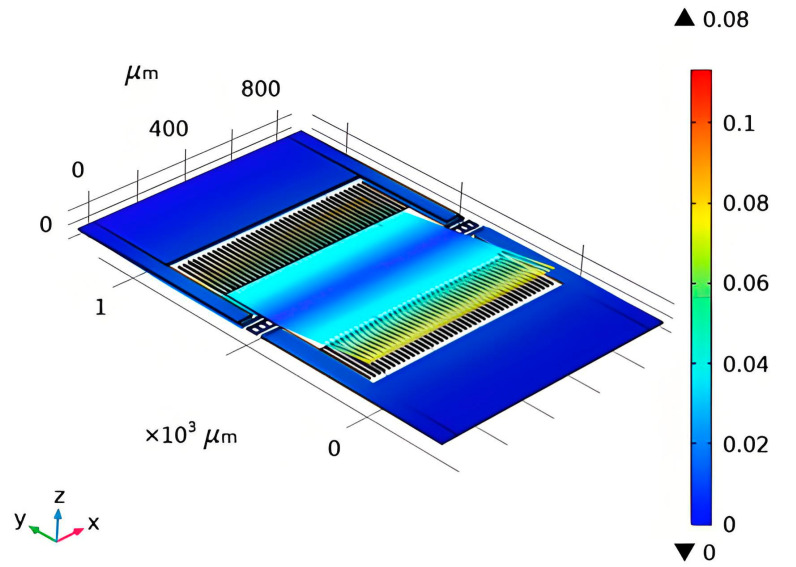
Electric field sensing structure of MEMS-sensitive module.

The structure of the MEMS-sensitive module is shown in Figure 1. The staggered comb structure design is adopted to efficiently utilize the internal space of the component cavity, increase the coupling effect between the electrode and the electric field, and achieve a higher electric field response from the small-sized induction electrode, thus improving the sensitivity of the sensor. Under the finite element simulation conditions, the tooth end of the comb presents a stronger induced electric field intensity than other components, demonstrating the precise electric field measurement effect of miniaturized devices.

**Figure 2:**
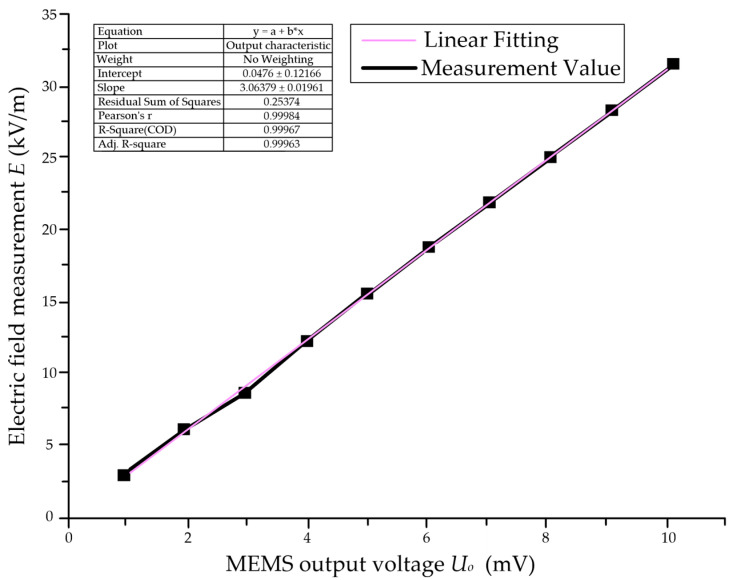
Input/output characteristic curve of MEMS electric field sensor.

In order to accurately find out the relationship between the output of the electric field sensor and the external electric field, the sensor was tested, and input and output characteristic curves of the electric field sensor were obtained (see Figure 2).

The input–output curves of the MEMS sensor were linearly fitted to obtain the linear fit equation intercept, slope, and linear fit. That is, *E* = 3.0638 *U_o_* + 0.0476, where *E* is the input electric field, *U_o_* is the output voltage of the electric field sensor, and the linear fit R^2^ is 0.9998; the closer to 1, the better the linear fit. According to the input and output characteristics of the sensor under the action of power frequency, it can be concluded that the sensor has a good linear input and output relationship in the range of 5 kV/m~30 kV/m. The sensor can be calibrated according to this linear relationship, and it can be used to realize the electric field measurement in the near-electricity early warning device.

### 2.2. Measurement Principle of the Ultrasonic Distance Sensor

**Figure 3:**
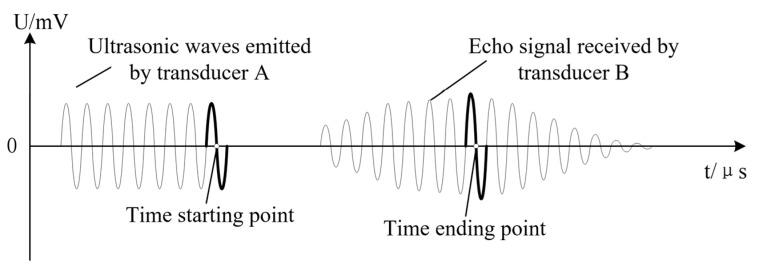
A schematic diagram of the ultrasonic crossing time.

The principle behind the distance measurement performed by the ultrasonic distance sensor involves the continuous emission of ultrasonic waves by the transmission transducer, which subsequently receives the echo signals reflected from the target being measured [21,22]. The sensor calculates the distance to the target by detecting the ultrasonic signals and utilizing the speed of ultrasonic signal propagation, denoted as *c*.

(5) $$d=\frac{1}{2}c\Delta T$$

where c is the propagation speed of the ultrasonic wave in the current environment, and ΔT is the crossing time of the ultrasonic signal transmission. In cases with fixed environmental parameters, the test accuracy of the target distance *d* is directly related to the determination of the crossing time (see Figure 3).

**Figure 4:**
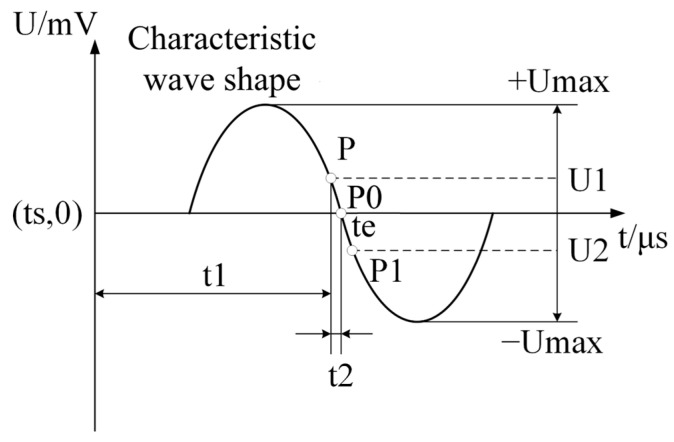
Schematic diagram of the characteristic waveforms.

First, a point-by-point comparison of the A/D sampling points is performed and the maximum value of the sampling point is identified to determine the period of the largest amplitude of the characteristic wave. A characteristic wave waveform diagram is shown in Figure 4. Second, we determine the crossing time corresponding to each sampling point before the zero point, P0 (P and P1). Clearly, the P sampling value is greater than 0, and the P1 sampling value is less than 0. Finally, using the time corresponding to P and P1 as a benchmark, the moment corresponding to P0 can be accurately calculated by the subdivision insertion algorithm, and then, the distance between the sensor and the measured target is calculated according to Equation (5).

### 2.3. Double-Criterion Signal Integrated Detection Circuit

**Figure 5:**
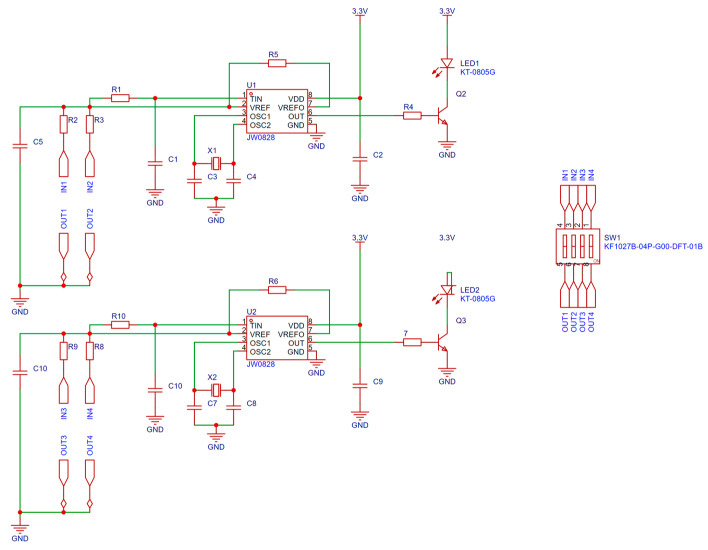
Test circuit design.

The detection of electric field and distance signals is primarily achieved through an integrated detection circuit, as shown in Figure 5. The signals collected by the electric field and distance sensors pass through an RC filter circuit composed of components such as C1, C10, R1, and R10. Capacitors and resistors exhibit a “trapping effect” on signals outside the resonant frequency, effectively filtering out high-frequency electric fields and distance signals while allowing the filtered signal to pass to the second pin of Q1 and Q3. After processing by Q1 and Q3, the signal outputs a square wave at a frequency of 4 kHz, which is utilized for transmitting electric field and distance information.

To mitigate the impact of temperature variations on circuit performance, a self-check circuit is integrated to ensure stability under diverse conditions. The dial code switch SW2 allows for the adjustment of resistances R, R3, Rg, and Ro, enabling a change in the circuit’s operational state and facilitating the transition between ‘test’ and ‘silent’ modes. The signal is output from pins 6 of transistors Q1 and Q3, and after amplification by Q2 and Q4, it is fed into LED1 and LED2 for indication.

The key to the stability and accuracy of the detection circuit lies in the application of the quartz oscillator. A quartz oscillator can produce mechanical vibration spontaneously under the action of the voltage signal at a specific frequency. With its unique mechanical and electronic characteristics, the oscillator enables the detection circuit to operate reliably in environments characterized by a high voltage, a high current, and strong electromagnetic interference, providing an accurate reference signal for the system and accurately identifying the electric field signal at a frequency close to 50 Hz.

The stability and accuracy of the detection circuit are fundamentally dependent on the application of the quartz oscillator. A quartz oscillator can spontaneously produce mechanical vibrations when subjected to a voltage signal at a specific frequency. Due to its unique mechanical and electronic characteristics, the oscillator allows the detection circuit to function reliably in environments characterized by high voltage, high current, and strong electromagnetic interference. This ensures the provision of an accurate reference signal for the system while effectively identifying the electric field signal at a frequency close to 50 Hz.

## 3. Preparation and Judgment Standard of Double Judgment of Near-Electricity Early Warning Device

### 3.1. Simulation of Near-Electricity Environment in Substation

In terms of double judgment, according to the standard for near-electricity early warning devices (reference GB 26860-2011), the focus is on ensuring safe distances between workers and construction machinery [23]. The minimum safety distance data, serving as safety red lines, is set to the near-electricity early warning distance alarm threshold. This consideration takes into account the operational behavior of workers and large mechanical devices, based on the first threshold (moderately relaxed thresh-old) and the distance alarm secondary threshold, as shown in Table 1.

**Figure 6:**
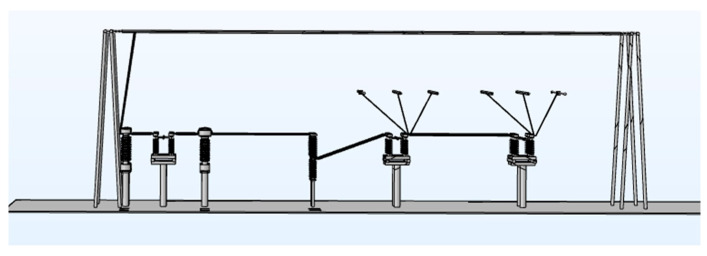
A 3D model of the main transformer in the 220 kV substation.

**Figure 7:**
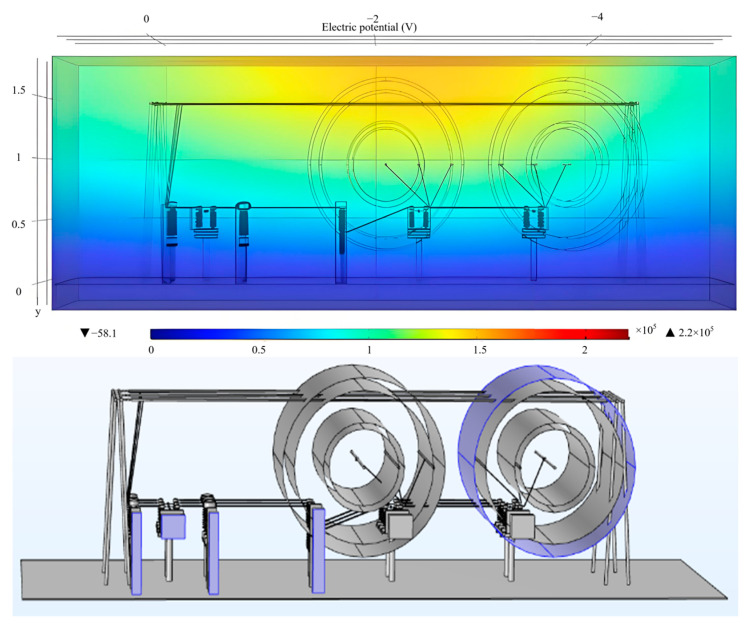
Potential nephogram distribution and measuring point distribution of main transformer bay in 220 kV substation.

In order to obtain the electric field intensity value at the two-stage distance threshold, as the electric field alarm threshold, this paper studies the judgment standard of the proposed near-electricity early warning device through COMSOL simulation and selects the typical operation scene of a 220 kV substation for electric field simulation calculation, as shown in Figure 6, analyzing the spatial electric field distribution by simulation (see Figure 7).

To obtain the electric field intensity value at the two-stage distance threshold, designated as the electric field alarm threshold, this paper investigates the judgment criteria of the proposed near-electricity early warning device through Comsol simulation. It selects a typical operational scenario of a 220 kV substation for electric field simulation calculations, as shown in Figure 6, and analyzes the spatial electric field distribution through simulation (see Figure 7).

**Figure 8:**
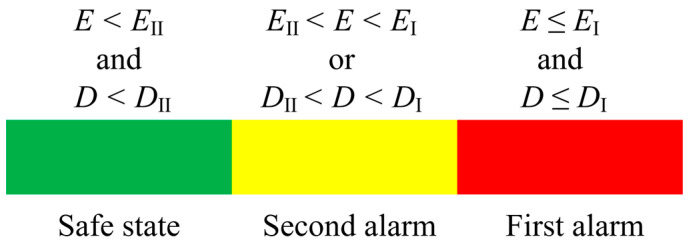
Multi-level alarm renderings of both criteria.

Considering the dense distribution of electrical equipment in the main transformer inlet line interval of the 220 kV substation (specifically, the 9 m interval between the current transformer and the circuit breaker), the electric field on the front side of the equipment is detected. For the bus, the electric field intensity amplitude near phase B is low, while the intensities near phases A and C are high. Therefore, we select phases A and C, as well as the area near the bus, as measuring points. In Figure 8, the items depicted from left to right are as follows: (1) isolating switch, current transformer, circuit breaker; (2) isolating switch; (3) isolating switch; (4) bus (left); and (5) bus (right).

The secondary threshold distribution points are located 3.5 m and 7 m from the front (rear) side of the equipment, as well as at 1# bus A and 2# bus C. Table 2 presents the electric field amplitude values measured at various distances from the electrical equipment. The simulation results are shown.

In order to verify the accuracy of our COMSOL simulation, a test was carried out near each piece of equipment in the 220 kV substation. The field measurement results are shown in Table 3. It can be seen that the measured electric field value is not much different from the simulation results, which proves the rationality of the device’s distance threshold and electric field threshold settings.

To verify the accuracy of our COMSOL simulation, we conducted tests near each piece of equipment in the 220 kV substation. The results of the field measurements are presented in Table 3. It is evident that the measured electric field values closely align with the simulation results, thereby substantiating the rationale behind the device’s distance threshold and electric field threshold settings.

### 3.2. Double-Criterion Over-Limit Early Warning System

**Figure 9:**
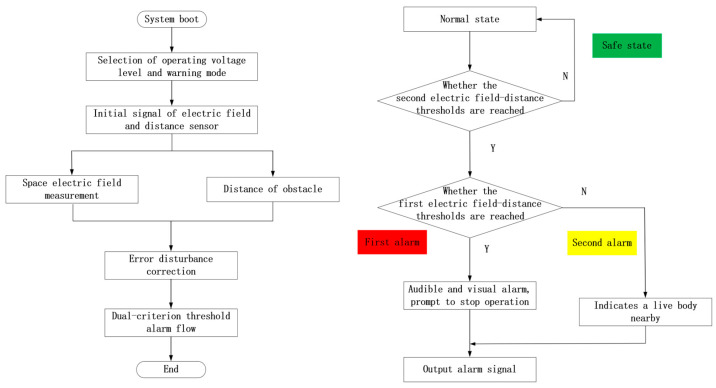
Flowchart of near-level warning of multi-level alarm.

Based on measurements of electric field and distance, it is essential to design the over-limit warning framework. In view of the problem that it is difficult to accurately judge the protected object near the charged body by a single criterion, The multi-level alarm threshold setting as shown in Figure 8 is adopted. The actual operation scenario is fully considered, and combined with the two criteria of electric field and distance, the early warning framework of the double criterion is constructed as shown in Figure 9.

After the warning system is activated, the working voltage level and warning mode (personnel operation warning and mechanical operation warning) are determined first, and then, the measurement of electric field and distance begins. By measuring the electric field strength of the operator or the machinery and the distance between the protected object and the surrounding live body, a comparison with the electric field alarm threshold and distance alarm threshold for all levels is performed to determine whether the alarm and the alarm level are needed.

After the warning system is activated, the working voltage level and warning mode (personnel operation warning and mechanical operation warning) are first determined. Subsequently, the measurement of the electric field and distance begins. By measuring the electric field strength of the operator or machinery and the distance between the protected object and any surrounding live bodies, a comparison is made with the electric field alarm threshold and distance alarm threshold for all levels to determine whether an alarm is necessary and what the alarm level should be.

In terms of software design, the system is initialized, and the double-criterion data are verified to ensure the high efficiency and accuracy of near-electricity measurement and distance quantity calculation. After selecting the safety threshold in line with the actual measurement, the system corrects the electric field and distance measurement disturbance, extracts the effective signal, and determines the corresponding alarm level based on the real-time comparison results between the two. Depending on various measurement scenarios, the distance measurement threshold can be relaxed in rainy and foggy environments to mitigate the impact of inaccurate distance criteria. In situations with a dense distribution of multiple conductors, the electric field threshold can be increased to counteract the interference caused by electric field coupling. The system is capable of adjusting the weights of the double criteria to achieve optimal output in complex environments.

The specific alarm effect is shown in Figure 8. The electric field measurements are denoted as E, while the distance measurements are represented as D. The primary and secondary electric field alarm thresholds are labeled EI and EII, respectively, and the primary and secondary distance alarm thresholds are designated as DI and DII, respectively.

### 3.3. Hardware Structure of Dual-Judgment Near-Electricity Warning Device

**Figure 10:**
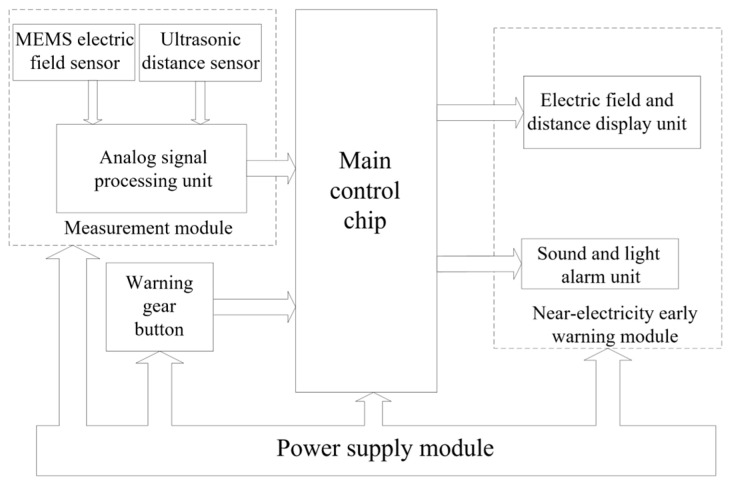
Hardware composition structure diagram of the near-power early warning device.

The hardware system of the near-electricity warning device comprises multiple functional modules, including an electric field measurement module, a distance measurement module, a master control chip, an early warning gear button, an alarm display module, and a power supply module, as shown in Figure 10.

The electric field measurement module and the distance measurement module are the core components of the entire device, responsible for real-time monitoring of the electric field strength surrounding the operator or machinery, as well as its position relative to the charged body. The measurement module employs the previously mentioned dual-sensor design, enabling it to detect changes in spatial electric field intensity and operational spacing in real time, and to transmit the data to the main control chip via the analog-to-digital conversion interface.

As the core control unit of the device, the main control chip is responsible for data processing and alarm logic judgment, comparing the set primary and secondary alarm thresholds. When the measured value exceeds the specified range, the main control chip triggers the alarm signal of the corresponding level and transmits the data to the early warning module.

The warning module comprises an acoustic and optical alarm unit alongside a data display unit. The acoustic and optical alarm unit delivers real-time alarm feedback through a buzzer and a bright LED indicator, while the data display unit visually presents the current electric field intensity and distance value.

**Figure 11:**
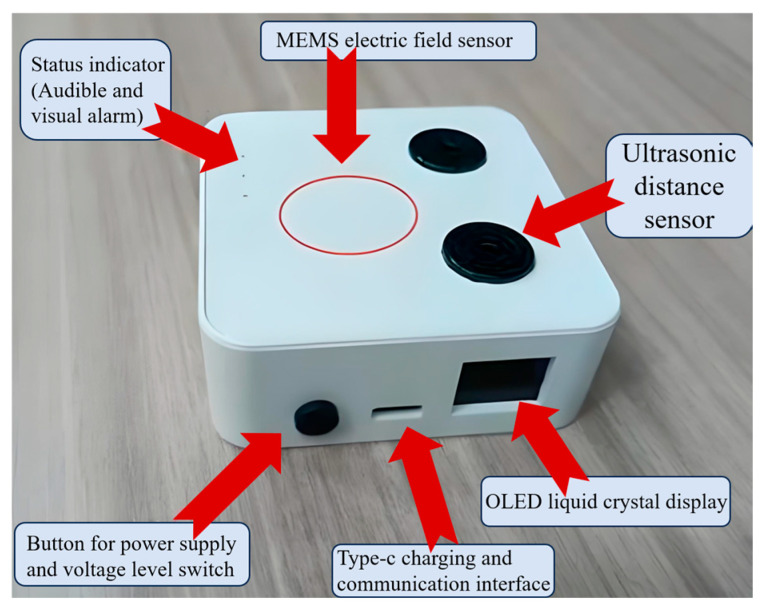
Prototype of near-electricity early warning device.

Figure 11 shows a prototype of the double electric early warning device, which adopts a portable design with integrated packaging, meaning it is easy to carry and install. It can also realize embedded integration with the portable safety equipment of the staff, which meets the development requirements of lightweight and intelligent power safety equipment, and is suitable for a variety of high-voltage working environments.

Figure 11 presents a prototype of the double electric early warning device, which features a portable design with integrated packaging, facilitating ease of transport and installation. Additionally, it enables embedded integration with the portable safety equipment used by personnel, aligning with the development requirements for lightweight and intelligent power safety equipment, and is suitable for various high-voltage working environments.

## 4. Field Application of Double-Judgment Short-Power Early Warning Device

In this study, the circuit breaker and the three-phase line in the operating environment were selected as the experimental object, and the practical application effect of the traditional YJ-AM-3 (Moufu Trading Company, Ltd., Nanjing, China) and the dual-power warning device was compared. The experiment verifies the alarm accuracy of the double-judgment power warning device by simulating the field power operation.

**Figure 12:**
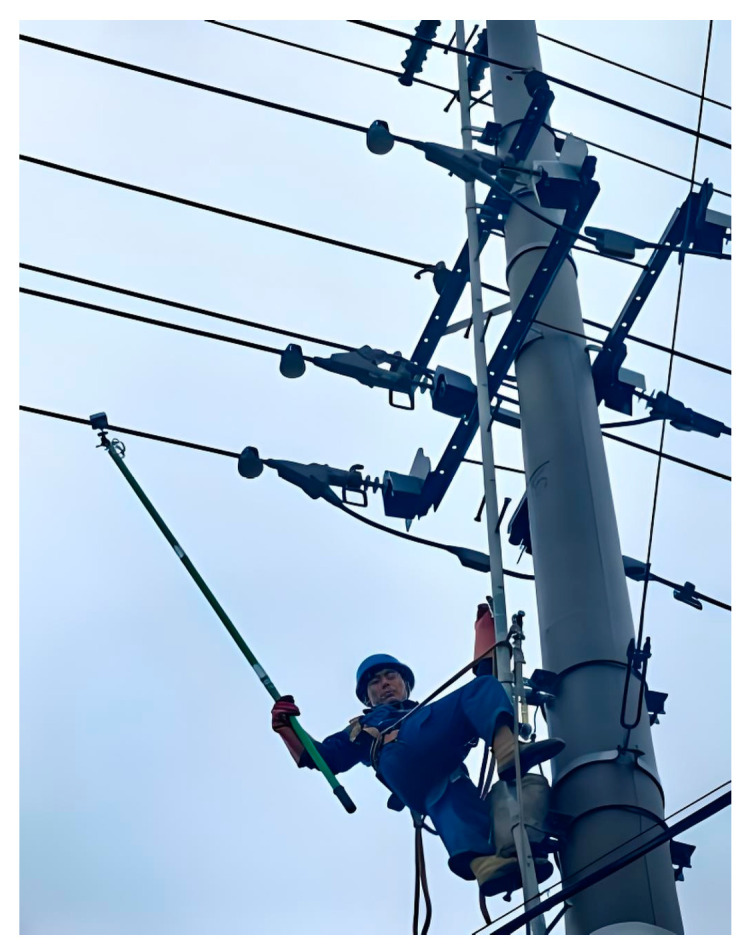
Field test diagram of near-electricity warning device.

Figure 12 illustrates a field test diagram of the near-electricity warning device. During the experiment, the warning device was affixed to an insulation rod, and a laser rangefinder was employed to measure the real-time distance between the warning device and the target equipment. The experimenter held the insulation pole with the warning device and gradually approached the target equipment from a distance within the operating space to observe the alarm responses of both warning devices.

**Figure 13:**
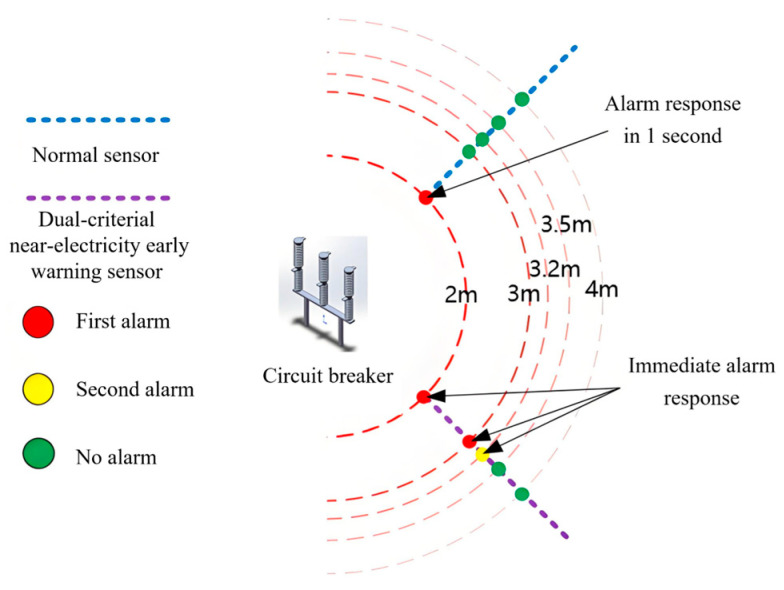
Alarm situation of double-warning devices in the operating environment of a 220 kV substation.

The double-warning alarm situation of the device in the operating environment of a 220 kV circuit breaker is shown in Figure 13. As the measurement distance decreases, the early warning level of the double-criterion device gradually increases, whereas the YJ-AM-3 triggers an alarm only at the test limit distance.

In the field test, the double-criterion sensor exhibited enhanced alarm sensitivity and a quicker response speed. The specific electric field output and alarm conditions of the sensor are presented in Table 4. In this table, sensor 1# refers to a standard YJ-AM-3 sensor, while sensor 2# denotes the double-criterion near-electricity sensor developed for this project.

The traditional YJ-AM-3 near-electricity early warning device is unable to distinguish between first and second level alarms, and it does not provide real-time readings of electric field values and distance measurements. The missing alarm phenomenon occurs in the 220 kV personnel safe distance scene, and the alarm response speed is slow, and the over-threshold strong field information cannot be fed back in time.

The double-criterion near-electricity warning device can measure both the electric field strength and the distance between the operator and live equipment within 0.1 s. Its alarm features, based on the criteria of electric field and distance, enhance accuracy. Unlike traditional sensors, which remain silent, the double-criterion sensor effectively distinguishes threat levels and triggers an alarm at distances of 3.2 m and 3 m. By setting different alarm thresholds, the double-criterion system effectively minimizes the occurrence of false alarms and missed alarms.

**Figure 14:**
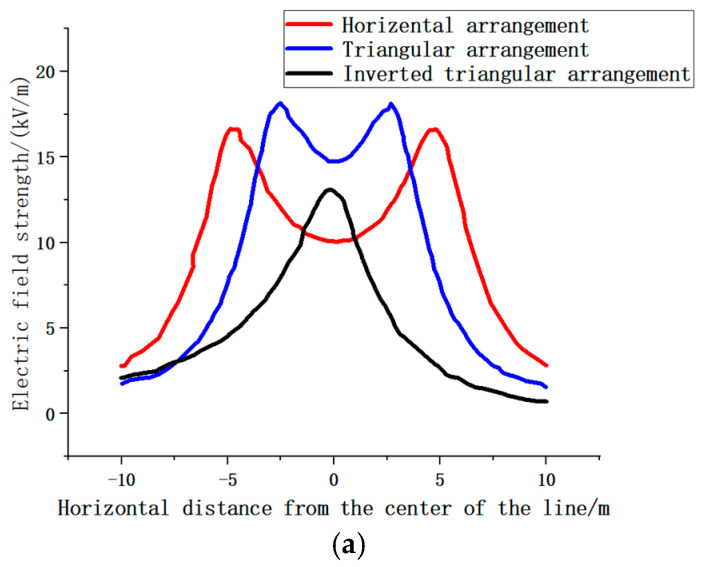

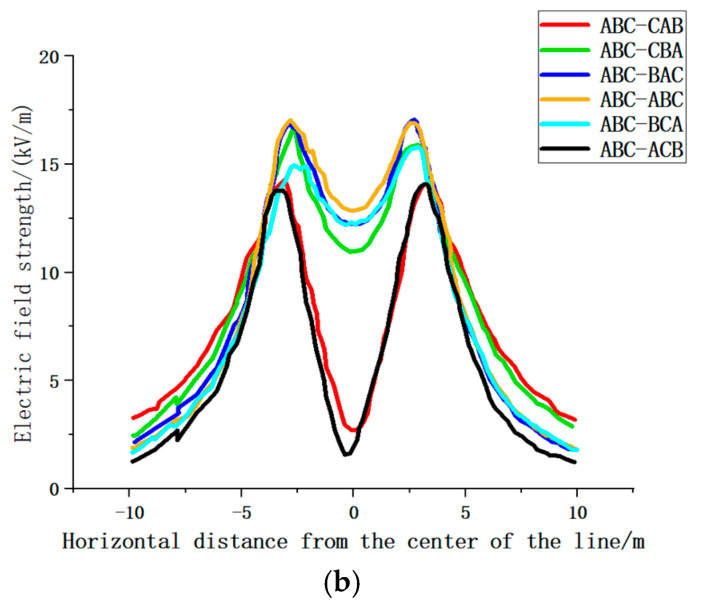
Field strength under single-loop transmission lines with different arrangement modes tested by warning device. (**a**) Field strength under single-loop transmission lines with different arrangement modes. (**b**) Field strength under dual-loop transmission lines with different phase sequence modes.

Figure 14 illustrates the distribution of electric field intensity at the terminal output at various distances from the three-phase 220 kV line. A notable negative correlation exists between the near-end electric field and the distances of the two-phase lines, A and C. Additionally, the peak value of the electric field strength of phase B is significantly lower than that of the other two phases. This reduction is attributed to the counteracting effect resulting from the superposition of the electric fields of phases A and C. Overall, the observed trends align with the simulation results.

Under the two-level alarm condition corresponding to the 220 kV voltage level, the system can respond quickly and issue the corresponding alarm correctly. In addition, the measurement accuracy of the alarm sensor enables the double-criterion near-electricity early warning device to flexibly respond to different environmental conditions and meet the increasingly complex safety needs of the smart substation. The rapid response rate and high accuracy of the device provide a higher level of safety guarantee for substation operation.

Under the two-level alarm condition corresponding to the 220 kV voltage level, the system can respond quickly and issue the appropriate alarm accurately. Furthermore, the measurement accuracy of the alarm sensor allows the double-criterion near-electricity early warning device to flexibly adapt to various environmental conditions, thereby addressing the increasingly complex safety requirements of the smart substation. The rapid response rate and high accuracy of this device offer an enhanced level of safety assurance for substation operations.

## 5. Conclusions

In this paper, a double-criterion near-electricity warning device based on electric field and distance measurement has been proposed. This device effectively addresses the issues associated with single-criterion systems, including a high false alarm rate and inadequate operational distance guarantees in traditional near-electricity warning technology.

The non-contact electric field measurement method utilizing MEMS sensors was investigated. This method integrates a dual-sensitive input mode for electric field and distance, enabling the extraction of measurement signals and clutter filtering through a conditioning detection circuit. Additionally, it incorporates a highly energy-efficient design that allows for mode switching. Through simulation-based research on the electric field environment in the vicinity of the main converter line at the substation, a double-criterion early warning system was developed to provide safety state feedback and instantaneous alarm information within 1 s in the substation environment.

The measurement of the near-end electric field value was accurate during the 220 kV line test, with no erroneous or missing reports. To enhance the reliability of near-electricity early warning technology, the device will undergo electrical inspection in environments with varying humidity, such as rainy and snowy conditions. Further tests will be conducted to verify the effectiveness of the device’s environmental interference shielding capabilities.

## Figures and Tables

**Table 1:** Setting of safety distance threshold for substation operators and machinery.

Requirement	Operators’ Safety Distance/m	Safety Distance of Working Machinery/m
Level I threshold	3	6
Secondary threshold	3.5	7

**Table 2:** Electric field amplitude (kV/m) values generated at different distances from the 220 kV substation. The results were derived by simulation.

Electric Accessory	3 m	3.5 m	6 m	7 m
1# Isolation switch	10.9	10.1	7.5	6.9
current transformer	9.8	9.0	7.3	6.8
circuit breaker	9.7	9.4	8.1	7.6
2# Isolation switch	12.4	12.0	9.8	8.9
3# Isolation switch	12.6	12.0	9.5	8.6
1# Bus A phase	6.3	6.0	9.9	9.5
2# Bus C phase	9.7	5.0	9.6	8.4

**Table 3:** Field measurement results regarding 220 kV substation (kV/m).

Electric Accessory	3 m	3.5 m	6 m	7 m
1# Isolation switch	11.1	9.8	7.8	7.2
current transformer	10.2	9.3	7.8	7.1
circuit breaker	10.0	9.5	7.9	7.6
2# Isolation switch	11.7	11.5	9.0	7.9
3# Isolation switch	12.0	11.4	9.2	8.0
1# Bus A phase	8.0	7.1	9.0	8.2
2# Bus C phase	8.2	6.2	5.0	4.2

**Table 4:** Alarm situation of near-electricity early warning devices in 220 kV substation operating environment.

	Distance Between Device and Circuit Breaker/m									
	4		3.5		3.2		3		2	
Types of Sensors	1#	2#	1#	2#	1#	2#	1#	2#	1#	2#
Field Strength Display/(kV/m)	/	14	/	15.5	/	16.8	/	17.5	/	18.5
Alarm Condition	Not Satisfied	Not Satisfied	Not Satisfied	Not Satisfied	Not Satisfied	Second Alarm Satisfied	Not Satisfied	First Alarm Satisfied	Satisfied	First Alarm Satisfied
Device alarm effect	No Alarm	No Alarm	No Alarm	No Alarm	No Alarm	Second Alarm	No Alarm	First Alarm	Alarm	First Alarm
Alarm Response Delay	/	/	/	/	/	Less than 0.1 s	/	Less than 0.1 s	1 s	Less than 0.1 s

## Data Availability

Data are contained within the article.
